# Prenatal-postnatal integrated management model improves outcomes of neonatal cardiac surgery in critical congenital heart disease: a retrospective cohort study

**DOI:** 10.1186/s12887-025-06378-x

**Published:** 2026-01-05

**Authors:** Yuekun Sun, Yiping Han, Gang Li, Yongtao Wu, Jun Yan, Qiang Wang

**Affiliations:** 1 Department of Pediatric Cardiac Center, Beijing Anzhen Hospital, Capital Medical University, Beijing, China; 2 Capital Medical University, Beijing, China

**Keywords:** Critical congenital heart disease, Prenatal-postnatal integrated management model, Prenatal diagnosis, Immediate postnatal surgery, Neonate

## Abstract

**Background:**

Neonates with CCHD remain at high risk for mortality. This study aimed to evaluate the impact of the prenatal-postnatal integrated management model (PPIMM) on perioperative outcomes and mortality in neonates with CCHD, and to identify factors associated with mortality.

**Methods:**

This retrospective cohort study enrolled 274 neonates with CCHD undergoing cardiac surgery from January 2018 to August 2024. Patients were categorized into the PPIMM group (*n* = 140) and the non-PPIMM group (*n* = 134). The primary outcome was all-cause mortality, including operative mortality and late mortality. The secondary outcome was unplanned reoperation. Kaplan–Meier survival and Cox regression analyses were performed.

**Results:**

The PPIMM group underwent surgery at a younger age (8.00 vs. 16.00 days, *P* < 0.001), with lower rates of preoperative intubation (11.43% vs. 20.90%, *P* = 0.047), emergent procedures (5.71% vs. 14.18%, *P* = 0.032), and operative mortality (5.71% vs. 13.43%, *P* = 0.036) compared to the non-PPIMM group. The median follow-up was 20.70 months (IQR 9.00–30.30) in the PPIMM group and 20.40 months (IQR 7.00–37.50) in the non-PPIMM group. Late mortality (3.57% vs. 4.48%, *P* = 0.702) and unplanned reoperation rates (*P*>0.05) were comparable between the PPIMM and non-PPIMM groups. Kaplan–Meier analysis showed a significant survival advantage for all-cause mortality in the PPIMM group (log-rank *P* = 0.038). Twelve neonates underwent immediate postnatal surgery, with no deaths or reoperations during a median follow-up of 13.00 months. PPIMM was a protective factor for operative mortality in the overall cohort, and prenatal diagnosis was protective within the non-PPIMM subgroup, while preoperative intubation, postoperative ECMO use, and elevated lactate level at 24 h postoperatively were risk factors.

**Conclusions:**

PPIMM was associated with earlier surgery, better preoperative status, and lower operative mortality in neonates with CCHD. In selected neonates, immediate postnatal surgery appeared feasible and was not associated with increased mortality. PPIMM and prenatal diagnosis were protective factors, whereas preoperative intubation, postoperative ECMO, and elevated lactate at 24 h postoperatively were risk factors for operative mortality.

**Trial registration:**

Retrospectively registered at ClinicalTrials.gov (NCT06768008), 2025-01-03.

**Supplementary Information:**

The online version contains supplementary material available at 10.1186/s12887-025-06378-x.

## Background

Congenital heart disease is one of the most common congenital anomalies worldwide, with an incidence of 4 to 50 per 1,000 live births [1, 2]. Among these, critical congenital heart disease (CCHD) accounts for about 20–25% of all CHD cases [3, 4]. The complexity of surgery and perioperative management both influence surgical outcomes in neonates with CCHD. Despite advances in surgical techniques in recent years, CCHD still carries a high mortality rate, with reports indicating neonatal mortality rates ranging from 7.4% to 15.1% [5, 6]. In neonates with CCHD, the transition from fetal to postnatal circulation often leads to significant hemodynamic instability and rapid worsening of clinical status. Any delay in intervention may result in poor outcomes. However, nearly half of infants are diagnosed postnatally and are often delivered at hospitals lacking neonatal surgical capabilities, requiring postnatal transfer, which ultimately leads to treatment delays [7, 8]. Many infants arrive at the hospital in compromised condition, such as severe hypoxemia, arrhythmias, or necrotizing enterocolitis, all of which can adversely affect prognosis [9, 10].

The prenatal-postnatal integrated management model (PPIMM) provides a coordinated approach that integrates prenatal diagnosis and counseling, planned delivery, and neonatal surgery into a continuous, well-coordinated process of care [11, 12]. Under PPIMM, risk assessment is conducted before birth, delivery is arranged at specialized centers capable of performing CCHD surgery, and affected infants receive timely, individualized interventions based on their specific risk profiles after delivery. PPIMM is intended to improve outcomes in neonates with CCHD. However, evidence regarding its effectiveness in reducing neonatal surgical mortality remains limited.

This study systematically analyzed postoperative clinical outcomes in neonates with CCHD managed with or without PPIMM, and compared the outcomes between two different time periods. Moreover, the feasibility and safety of immediate postnatal surgery under the PPIMM were evaluated. Additionally, multivariable analysis was conducted to identify independent risk factors for mortality. The findings of this study help clinicians optimize perioperative management and develop more individualized surgical strategies for neonates with CCHD, thereby improving patient outcomes.

## Methods

### Study population

**Fig. 1:**
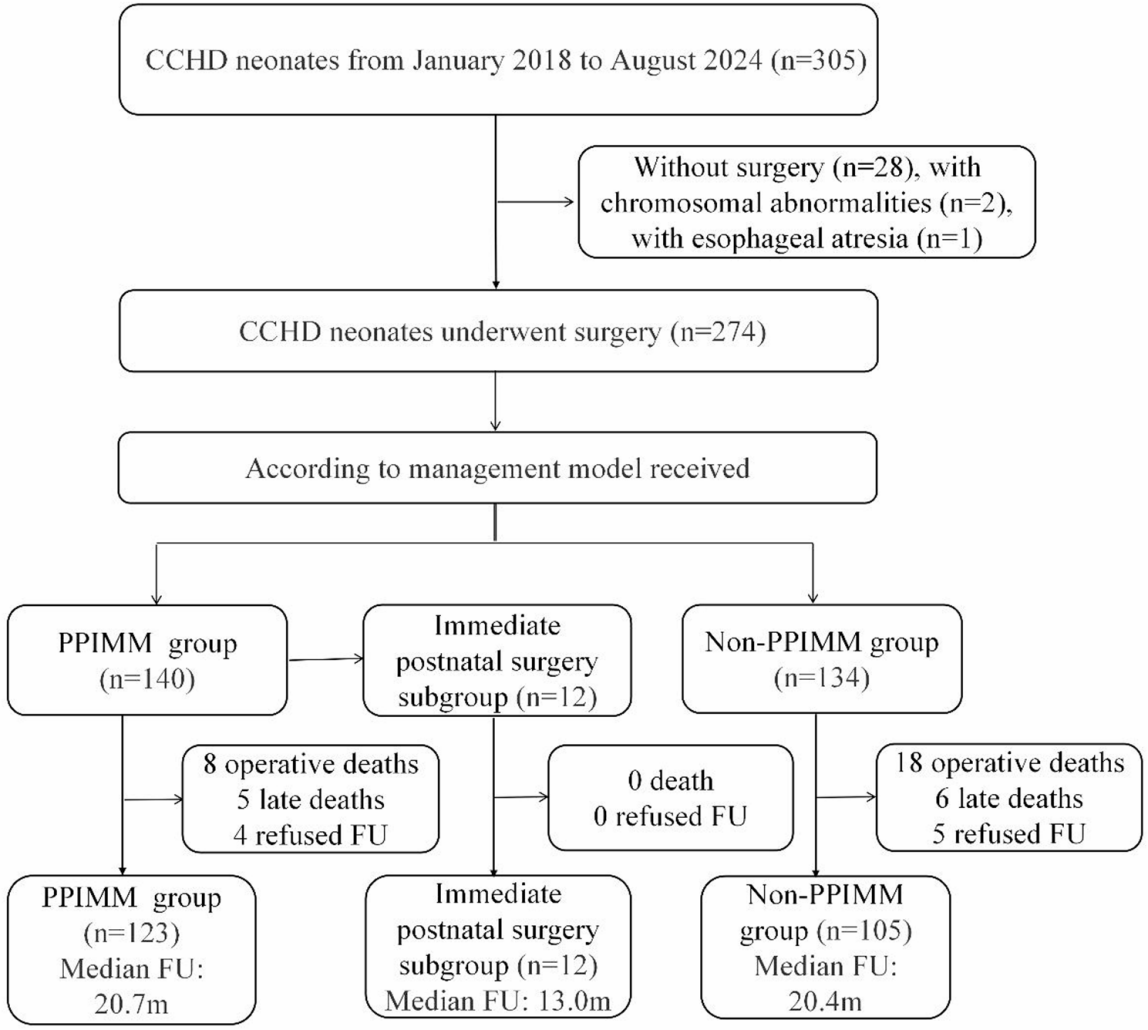
Flowchart of patient recruitment and follow-up. Abbreviations: PPIMM, Prenatal–postnatal integrated management model; FU, Follow-up; CCHD, Critical congenital heart disease; m, Months; n, Number of patients

This study consecutively enrolled neonates with CCHD who underwent cardiac surgery during the neonatal period at Beijing Anzhen Hospital between January 2018 and August 2024 (Fig. 1). CCHD was defined to include the following conditions: transposition of the great arteries (TGA), total anomalous pulmonary venous connection (TAPVC), interrupted aortic arch (IAA), coarctation of the aorta (COA), pulmonary atresia (PA), tetralogy of fallot (TOF), severe pulmonary stenosis, hypoplastic left heart syndrome, double outlet right ventricle, and other major life-threatening cardiac malformations. Inclusion Criteria were: (1) Diagnosis of CCHD; (2) Age < 28 days at the time of cardiac surgery; (3) Complete clinical data available. Exclusion criteria were: (1) chromosomal abnormalities; (2) severe, life-threatening non-cardiac malformations; and (3) failure to receive surgical treatment. Patients were categorized into two groups based on whether they received prenatal-postnatal integrated management at Beijing Anzhen Hospital: the PPIMM group and the non-PPIMM group. The PPIMM group included neonates who were prenatally diagnosed with CCHD and delivered at Beijing Anzhen Hospital. The non-PPIMM group consisted of neonates who were delivered at referring hospitals and subsequently transferred to our center for treatment, irrespective of their prenatal diagnostic status. The key differences in perioperative management between the two groups are summarized in Supplementary Table 1. This study was conducted in accordance with the Declaration of Helsinki, approved by the Ethics Committee of Beijing Anzhen Hospital (Approval No.: ZD2024010), and registered at ClinicalTrials.gov (Registration No.: NCT06768008). Written parental informed consent was obtained for all participants.

### Perioperative management protocols

#### PPIMM group (Supplementary Fig. 1)

Neonates in the PPIMM group were diagnosed prenatally with CCHD, either diagnosed externally or at Beijing Anzhen Hospital. Following the initial diagnosis, serial fetal echocardiography was performed at regular intervals at our Maternal-Fetal Medicine Center to monitor both cardiac development and disease progression. Then a multidisciplinary team consisting of pediatric cardiologists, fetal echocardiography specialists, and obstetricians evaluated the fetal echocardiographic findings, assessed postnatal hemodynamic risk, and developed individualized perinatal management plans for delivery and initial treatment. After planned delivery, neonates were promptly admitted to the cardiac intensive care unit, where they underwent standardized preoperative monitoring and evaluation, including transthoracic echocardiography, electrocardiography, chest X-ray, and cardiac computed tomography as clinically indicated. Supportive interventions, such as oxygen supplementation, vasoactive agents, diuretics, and prostaglandin E₁ (for ductus-dependent CCHD), were provided when necessary.

For most patients, surgery was scheduled at the earliest clinically appropriate time following completion of the preoperative assessment. For selected neonates, the decision for immediate postnatal surgery was made during multidisciplinary prenatal consultations. On the day of delivery, the multidisciplinary team was on standby and fully prepared. The neonate was delivered and, after initial assessment, was immediately handed over by the obstetrician to the pediatric cardiac surgeon. The surgeon completed hospital registration, the pediatric echocardiographer performed cardiac ultrasound, and the transfusion service conducted cross-matching. All preoperative assessments were scheduled to be completed within two hours of birth, after which the neonate proceeded to anesthesia and underwent surgical repair (Supplementary Fig. 2).

#### Non-PPIMM group

Neonates in the non-PPIMM group were born at referring hospitals, diagnosed with CCHD either prenatally or postnatally, and were transferred to our center for further management via a regionally coordinated transfer system. Upon arrival, neonates were promptly admitted to the cardiac intensive care unit, where they underwent the same standardized preoperative evaluation and perioperative support as the PPIMM group, followed by neonatal surgical intervention. During the study period, surgical repair was the primary strategy for neonatal CCHD management. Surgical indications and procedures were determined solely by each patient’s anatomical and clinical characteristics and were consistent across both groups.

### Data collection and follow-up

Data were retrospectively retrieved from the electronic medical record system of Beijing Anzhen Hospital and verified independently by two investigators. Collected variables included demographic, preoperative, surgical, postoperative and follow-up data. Demographic information included sex, age, weight, preterm birth, gestational age, mode of delivery, in vitro fertilization, and twin pregnancy. Preoperative information included diagnosis, jaundice, arrhythmia, use of vasoactive drugs and prostaglandin E1, mode of respiratory support, and cardiopulmonary resuscitation. Surgical information included emergency surgery, Society of Thoracic Surgeons–European Association for Cardio-Thoracic Surgery (STAT) category, cardiopulmonary bypass (CPB) time, and aortic cross-clamp (ACC) time. Surgical complexity was assessed using the STAT mortality score [13]. Postoperative data included mortality, delayed sternal closure, extracorporeal membrane oxygenation (ECMO), peritoneal dialysis, arterial blood lactate level, vasoactive-inotropic score (VIS) and postoperative complications. VIS was used to quantify the level of hemodynamic support during the postoperative period [14]. Postoperative complications included pneumonia, chylothorax, wound infection, atelectasis, necrotizing enterocolitis, venous thrombosis, arrhythmias, permanent pacemaker implantation, capillary leak syndrome, stroke and hypoxic-ischemic encephalopathy.

Patients were followed up after discharge through outpatient visits and telephone interviews. All patients were scheduled for follow-up visits at 1 week, and at 1, 3, 6, and 12 months after surgery, and annually thereafter, which included echocardiography and electrocardiography. Follow-up data included mortality and reoperation occurring during the follow-up period. The primary outcome was all-cause mortality, encompassing both operative and late mortality. Operative mortality was defined as: (1) death occurring during the index hospitalization for surgery, even beyond 30 days postoperatively; (2) death occurring after discharge but within 30 days after surgery [5]. Late mortality was defined as death occurring beyond 30 days after surgery and not during the index hospitalization. The secondary outcome was unplanned reoperation. The composite endpoint was defined as the occurrence of either all-cause mortality or unplanned reoperation. Survival time was calculated from the date of initial surgery. For survival analysis, all deaths before discharge or during follow-up were considered events.

### Statistical analysis

Continuous variables were expressed as mean ± standard deviation (SD) or median (interquartile range, IQR) as appropriate, and compared using the independent samples t-test or Wilcoxon rank-sum test. Categorical variables were presented as frequencies (%) and compared using the Chi-square or Fisher’s exact test. Survival was assessed with Kaplan–Meier analysis and compared by the log-rank test. Associations with operative mortality were examined using univariable and multivariable Cox regression in the overall cohort and in each group separately. Variables with *P* < 0.10 in univariable analysis or deemed clinically relevant were included in multivariable models. Results were reported as hazard ratios (HRs) with 95% confidence intervals (CIs). The optimal postoperative lactate cutoff for predicting operative mortality was determined by receiver operating characteristic analysis using the Youden index. A two-sided *P* < 0.05 was considered statistically significant. Analyses were performed in R (version 4.3.2).

## Results

### CCHD subtype distribution

**Fig. 2:**
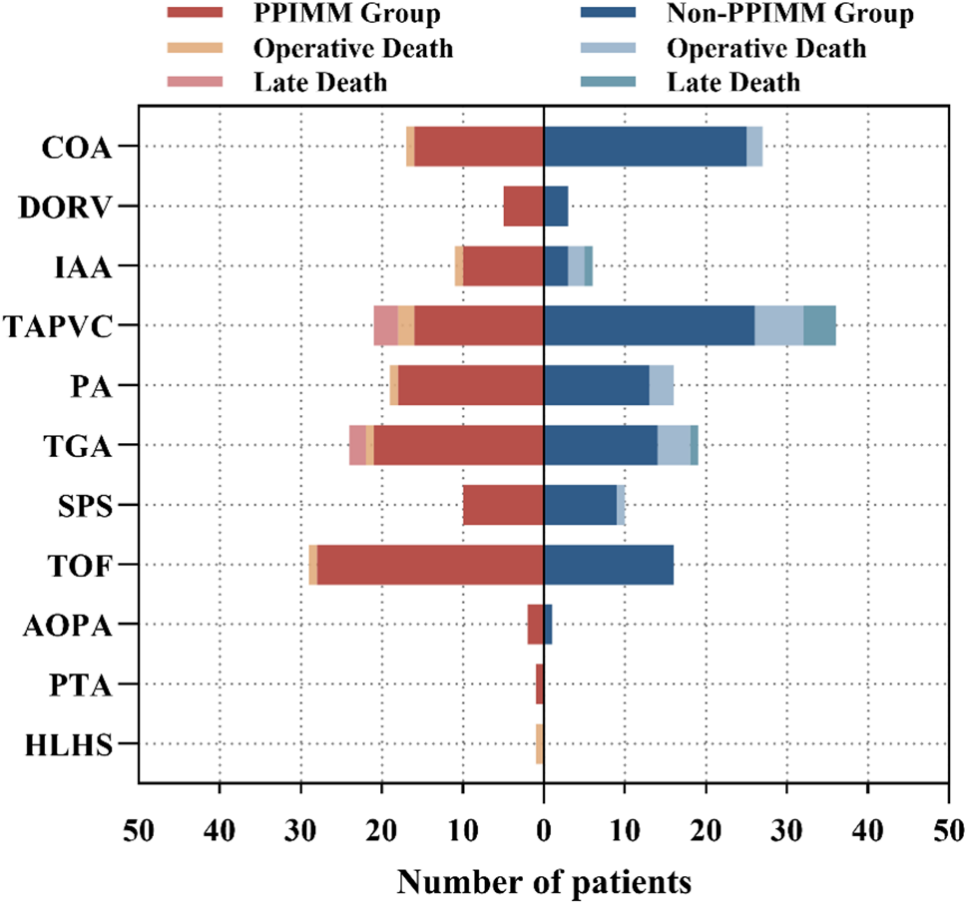
Distribution of disease categories and associated mortality in the PPIMM and non-PPIMM groups. Abbreviations: AOPA, Anomalous origin of pulmonary artery from ascending aorta; COA, Coarctation of the aorta; DORV, Double outlet right ventricle; HLHS, Hypoplastic left heart syndrome; IAA, Interrupted aortic arch; PA, Pulmonary atresia; PPIMM, Prenatal–postnatal integrated management model; PTA, Persistent truncus arteriosus; SPS, Severe pulmonary stenosis; TAPVC, Total anomalous pulmonary venous connection; TGA, Transposition of the great arteries; TOF, Tetralogy of fallot

This study included a total of 274 neonates with CCHD, comprising 140 cases (51.09%) in the PPIMM group and 134 cases (48.91%) in the non-PPIMM group (Fig. 2, Supplementary Table 2). Among all patients, the most common diagnoses were TAPVC (20.80%) and TOF (16.42%). In the PPIMM group, TOF (20.71%) and TGA (17.14%) were the predominant diagnoses, whereas TAPVC (26.87%) and COA (20.15%) were the most common subtypes in the non-PPIMM group. The proportion of TAPVC was significantly higher in the non-PPIMM group compared to the PPIMM group (26.87% vs. 15.00%, *P* = 0.023), while the differences in the distribution of other subtypes were not statistically significant (*P* > 0.05).

### Baseline characteristics

The rate of prenatal diagnosis was significantly higher in the PPIMM group than in the non-PPIMM group (100.00% vs. 52.99%; *P* < 0.001). Patients in the PPIMM group were admitted immediately after birth (0.00 days [IQR, 0.00–0.00] vs. 10.00 days [IQR, 4.00–15.75], *P* < 0.001), underwent surgery at a younger age (8.00 days [IQR, 3.00–14.00] vs. 16.00 days [IQR, 10.00–21.00], *P* < 0.001), and had a slightly lower weight at surgery (3.16 kg [IQR, 2.80–3.46] vs. 3.30 kg [IQR, 2.95–3.74], *P* = 0.012) compared to the non-PPIMM group. There were no statistically significant differences between the two groups in sex, gestational age, preterm birth, cesarean section, twin pregnancy, or in vitro fertilization (Table 1).

**Table 1:** Baseline characteristics between the PPIMM and non-PPIMM groups

Variable	PPIMM group(*n* = 140)	Non-PPIMM group(*n* = 134)	*P* value
Male sex [*n* (%)]	97(69.29%)	86(64.18%)	0.442
Gestational age, weeks	38.00(37.00,39.00)	39.00(38.00,39.00)	0.083
Premature infant [*n* (%)]	17(12.14%)	10(7.46%)	0.273
Cesarean section [*n* (%)]	101(72.14%)	84(62.69%)	0.123
Twin pregnancy [*n* (%)]	14(10.00%)	12(8.96%)	0.929
In Vitro Fertilization [*n* (%)]	14(10.00%)	6(4.48%)	0.127
Age at admission, days	0.00(0.00,0.00)	10.00(4.00,15.75)	< 0.001
Age at surgery, days	8.00(3.00,14.00)	16.00(10.00,21.00)	< 0.001
Weight at surgery, kg	3.16(2.80,3.46)	3.30(2.95,3.74)	0.012
Prenatal diagnosis [*n* (%)]	140(100.00%)	71(52.99%)	< 0.001
Preoperative jaundice [*n* (%)]	31(22.14%)	34(25.37%)	0.627
Preoperative arrhythmia [*n* (%)]	6(4.29%)	3(2.24%)	0.541
Preoperative ventilation mode [*n* (%)]			0.089
Spontaneous breathing	103(73.57%)	89(66.42%)	0.356
Non-invasive ventilation	21(15.00%)	17(12.69%)	0.387
Endotracheal intubation	16(11.43%)	28(20.90%)	0.047
Preoperative cardiopulmonary resuscitation [*n* (%)]	10(7.14%)	15(11.19%)	0.342
Preoperative vasoactive drug use [*n* (%)]	32(22.86%)	29(21.64%)	0.923
Preoperative prostaglandin E1 use [*n* (%)]	61(43.57%)	45(33.58%)	0.116
STAT category [*n* (%)]			0.988
STAT-1	2(1.43%)	2(1.49%)	-
STAT-2	47(33.57%)	42(31.34%)	-
STAT-3	34(24.29%)	31(23.13%)	-
STAT-4	56(40.00%)	58(43.28%)	-
STAT-5	1(0.71%)	1(0.75%)	-

*Abbreviations*: *PPIMM* Prenatal–postnatal integrated management model, *STAT* Society of Thoracic Surgeons–European Association for Cardio-Thoracic Surgery

Regarding preoperative clinical conditions, the rate of preoperative intubation was significantly higher in the non-PPIMM group than in the PPIMM group (20.90% vs. 11.43%, *P* = 0.047). There were no significant differences between the two groups in the incidence of jaundice, arrhythmias, cardiopulmonary resuscitation, vasoactive drug use, or prostaglandin E₁ administration.

### Surgical and postoperative outcomes

Regarding surgical procedures, the proportion of emergency surgeries was significantly lower in the PPIMM group compared to the non-PPIMM group (5.71% vs. 14.18%, *P* = 0.032), while there were no significant differences between the two groups in STAT categories, palliative surgery, the proportion of CPB, CPB duration, or ACC time (Table 2).

**Table 2:** Surgical characteristics and postoperative outcomes of the PPIMM and Non-PPIMM groups

Variable	PPIMM group(*n* = 140)	Non-PPIMM group(*n* = 134)	*P* value
Emergent procedure [*n* (%)]	8(5.71%)	19(14.18%)	0.032
CPB surgery [*n* (%)]	135(96.43%)	130(97.01%)	1.000
CPB time, min	140.00(106.00,165.00)	142.00(106.25,185.00)	0.287
ACC time, min	85.50 (65.00–110.25)	83.00 (59.25–109.50)	0.536
Palliation surgery[*n* (%)]	1(0.71%)	1(0.75%)	1.000
Postoperative mechanical Ventilation time, hours	96.75(57.87,179.00)	102.50(69.12,169.12)	0.878
Reintubation [*n* (%)]	10(7.14%)	10(7.46%)	1.000
Postoperative ICU stay, days	11.00(7.00,16.00)	10.00(6.25,13.00)	0.112
Delayed sternal closure [*n* (%)]	20(14.29%)	25(18.66%)	0.416
Peritoneal dialysis [*n* (%)]	21(15.00%)	26(19.40%)	0.420
ECMO [*n* (%)]	4(2.86%)	6(4.48%)	0.694
Highest VIS within 24 h after surgery	13.09(8.15,18.74)	11.40(8.07,16.18)	0.519
Lactate at ICU arrival, mmol/L	2.35(1.42,3.90)	2.50(1.37,3.82)	0.916
Lactate at 24 h after surgery, mmol/L	2.25(1.62,3.20)	2.70(1.80,4.33)	0.011
Complications [*n* (%)]			
Arrhythmias	13 (9.28%)	13 (9.70%)	1.000
Permanent pacemaker implantation	1(0.71%)	2(1.49%)	0.970
Chylothorax	2(1.43%)	1(0.75%)	1.000
Diaphragmatic paralysis	1(0.71%)	1(0.75%)	1.000
Wound infection	5(3.57%)	3(2.24%)	0.723
Atelectasis	13 (9.29%)	12 (8.96%)	1.000
Venous thrombosis	2(1.43%)	1(0.75%)	1.000
Necrotizing enterocolitis	1(0.71%)	0(0.00%)	1.000
Capillary leak syndrome	9(6.43%)	8(5.97%)	1.000
Stroke	1(0.71%)	2(1.49%)	1.000
Hypoxic-ischemic encephalopathy	0(0.00%)	1(0.75%)	1.000
Operative mortality [*n* (%)]	8(5.71%)	18(13.43%)	0.036
Unplanned reoperation during hospitalization [*n* (%)]	13 (9.28%)	10 (7.46%)	0.586
Follow-up period, months	20.70(9.00–30.30)	20.40(7.00–37.50)	0.945
Late mortality [*n* (%)]	5(3.57%)	6(4.48%)	0.702

*Abbreviations*: *ECMO* Extracorporeal membrane oxygenation, *CPB* Cardiopulmonary bypass, *ACC* Aortic cross-clamp, *PPIMM* Prenatal–postnatal integrated management model, *VIS* Vasoactive–inotropic score

Postoperative results showed that, compared to the non-PPIMM group, the PPIMM group had a significantly lower operative mortality (5.71% vs. 13.43%; *P* = 0.036) and a lower lactate level at 24 h postoperatively (2.25 [1.62–3.20] mmol/L vs. 2.70 [1.80–4.33] mmol/L; *P* = 0.011). No statistically significant differences were identified between the two groups in terms of unplanned reoperation during hospitalization, postoperative mechanical ventilation duration, reintubation, delayed sternal closure, peritoneal dialysis, ECMO use, postoperative complications, highest VIS within the first 24 h after surgery, lactate level at ICU arrival, or postoperative ICU length of stay (all *P* > 0.05). Comparing PPIMM with the prenatally diagnosed non-PPIMM group, PPIMM showed earlier admission and earlier surgery (both *P* < 0.05; Supplementary Table 3). Operative mortality was also lower with PPIMM in this comparison, but the difference was not statistically significant.

### Follow-up results

Among the 274 patients, 265 (96.71%) completed follow-up, with a median duration of 20.00 months (IQR, 8.00–33.00). The PPIMM and non-PPIMM groups had follow-up rates of 97.14% (136/140) and 96.27% (129/134), with median durations of 20.70 months (IQR, 9.00–30.30) and 20.40 months (IQR, 7.00–37.50), respectively.

A total of 11 late deaths (4.01%) occurred, including 5 (3.57%) in the PPIMM group and 6 (4.48%) in the non-PPIMM group. There was no significant difference in late mortality rates between the two groups (*P* = 0.702). The median age at death during the follow-up period was 9.00 months (IQR, 6.00–11.00 months). In the PPIMM group, deaths occurred in patients diagnosed with TAPVC (*n* = 3, 60.00%) and TGA (*n* = 2, 40.00%), whereas in the non-PPIMM group, patients who died had TAPVC (*n* = 4, 66.67%), TGA (*n* = 1, 16.67%), and IAA (*n* = 1, 16.67%). The primary causes of death were postoperative pulmonary vein obstruction (*n* = 5, 45.45%), heart failure (*n* = 3, 27.27%), severe pneumonia (*n* = 2, 18.18%), and necrotizing enterocolitis (*n* = 1, 9.09%).

A total of 13 patients (4.74%) underwent reoperation during the follow-up period, including 5 (3.57%) in the PPIMM group and 8 (5.97%) in the non-PPIMM group. The median age at reoperation was 9.00 months (IQR, 5.00–12.00 months). In the PPIMM group, reoperation was performed in patients with PA (*n* = 3, 60.00%) and IAA (*n* = 2, 40.00%), whereas in the non-PPIMM group, patients who underwent reoperation had diagnoses of TAPVC (*n* = 3, 37.50%), TOF (*n* = 2, 25.00%), PA (*n* = 1, 12.50%), TGA (*n* = 1, 12.50%), and COA (*n* = 1, 12.50%). There was no significant difference in reoperation rates during the follow-up period between the two groups (*P* = 0.350). The primary indications for reoperation were pulmonary artery stenosis (*n* = 7, 53.84%), recurrent pulmonary vein obstruction (*n* = 3, 23.08%), and aortic arch obstruction (*n* = 3, 23.08%). Among the 13 patients who underwent reoperation, one case of TAPVC died due to pulmonary vein obstruction, while the remaining 12 survived throughout the follow-up period. Additionally, a subgroup comparison of patients with TAPVC was performed between the PPIMM and non-PPIMM groups, with detailed information provided in Supplementary Table 4.

### Immediate postnatal surgery

In the PPIMM group, 12 neonates underwent immediate postnatal surgery: six with transposition of the great arteries with an intact ventricular septum and six with pulmonary atresia with an intact ventricular septum and right-ventricular hypoplasia. Surgical biventricular repair under CPB was initiated in all patients within two hours after birth. The median CPB time was 165.00 min (IQR: 136.50–172.25), and the median ACC time was 90.00 min (IQR: 75.75–113.50). During hospitalization, one patient (8.33%) underwent reintubation, one (8.33%) required delayed sternal closure, and one (8.33%) received peritoneal dialysis. All patients recovered and were discharged without mortality. Throughout a median follow-up of 13.00 months (IQR, 9.00–15.00), no mortality or unplanned reoperation was observed, indicating acceptable midterm outcomes in this cohort. Detailed information is provided in Supplementary Table 5.

### Survival analysis

**Fig. 3:**
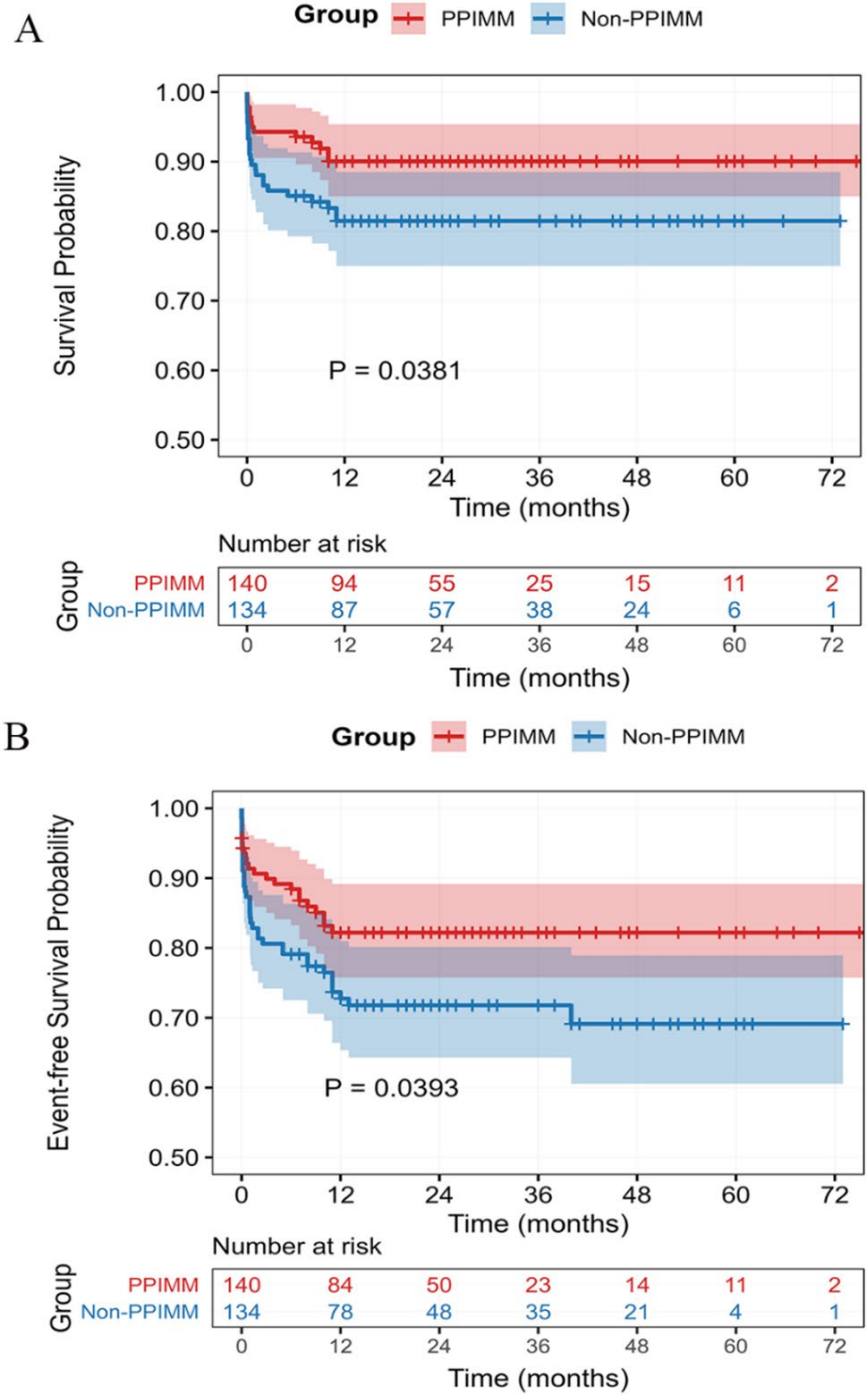
Kaplan-Meier survival analysis. (**A**) Survival curve based on all-cause mortality (operative mortality + late mortality). (**B**) Event-Free Survival Curve based on all-cause mortality or unplanned reoperation. Unplanned reoperation counted at any time during index hospitalization or follow-up. Survival time was calculated from the date of the index surgery. Numbers at risk are shown below each curve. Abbreviations: PPIMM, Prenatal–postnatal integrated management model

Kaplan–Meier curves revealed significantly improved survival in the PPIMM group compared to the non-PPIMM group based on all-cause mortality (Fig. 3A, *P* = 0.038). This survival benefit remained significant when assessed using a composite endpoint comprising all-cause mortality and unplanned reoperation (Fig. 3B, *P* = 0.039). Additionally, a subgroup analysis within the non-PPIMM group showed significantly higher survival in prenatally diagnosed patients compared with those diagnosed after birth (Supplementary Table 6; Supplementary Fig. 3, log-rank *P* = 0.014).

### Cox regression analysis

**Fig. 4:**
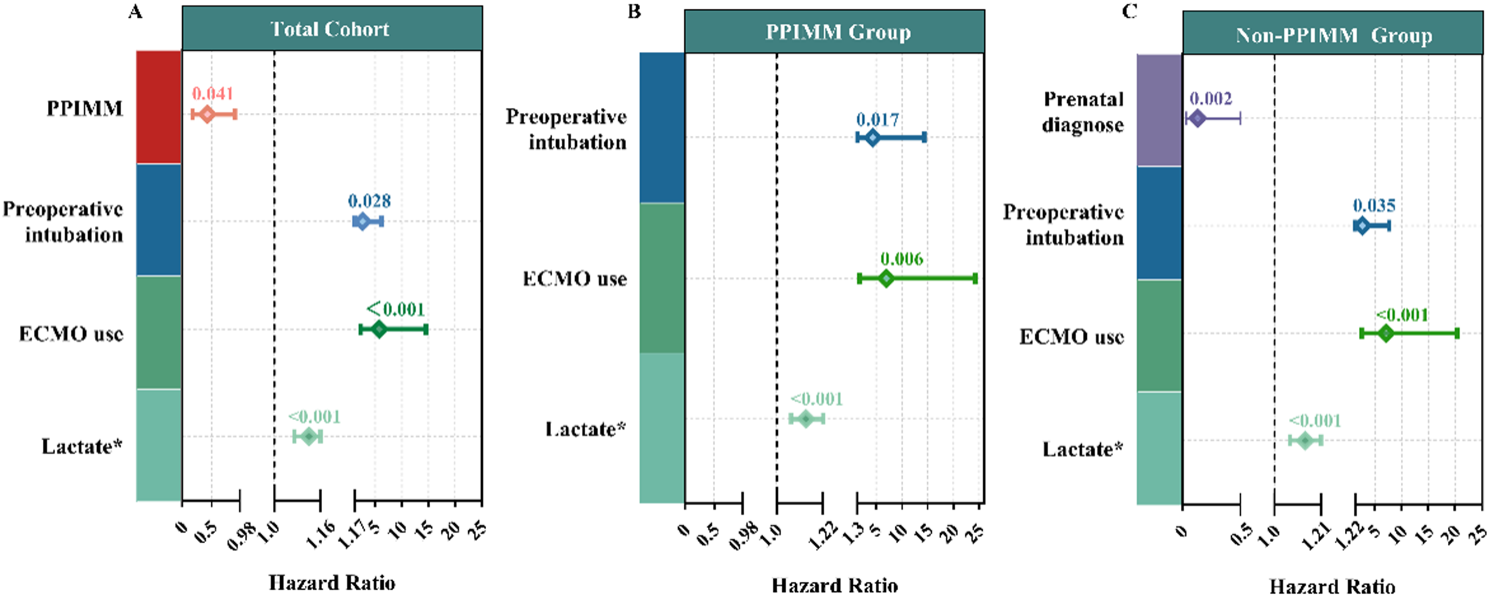
Forest plots of multivariable Cox regression models for operative mortality in (**A**) the Total cohort, (**B**) the PPIMM group, and (**C**) the non-PPIMM group. *Lactate refers to serum lactate level at 24 h after surgery. Abbreviations: PPIMM, Prenatal–postnatal integrated management model; ECMO, Extracorporeal membrane oxygenation

In the overall cohort, multivariable Cox regression analysis identified PPIMM as a significant protective factor against mortality (HR = 0.43, 95% CI: 0.18–0.90, *P* = 0.041). Preoperative intubation (HR = 2.63, 95% CI: 1.17–6.24, *P* = 0.028), postoperative ECMO use (HR = 5.76, 95% CI: 2.29–14.50, *P* < 0.001), and elevated lactate at 24 h postoperatively (HR = 1.12, 95% CI: 1.07–1.16 *P* < 0.001) were independent predictors of mortality (Fig. 4). The optimal cutoff value of 24-hour postoperative lactate for predicting operative mortality was 5.35 mmol/L (AUC = 0.74). Supplementary Table 7 presents univariable Cox results for the overall cohort and the PPIMM/non-PPIMM subgroups.

### Changes in outcomes: 2018–2021 vs. 2022–2024

**Fig. 5:**
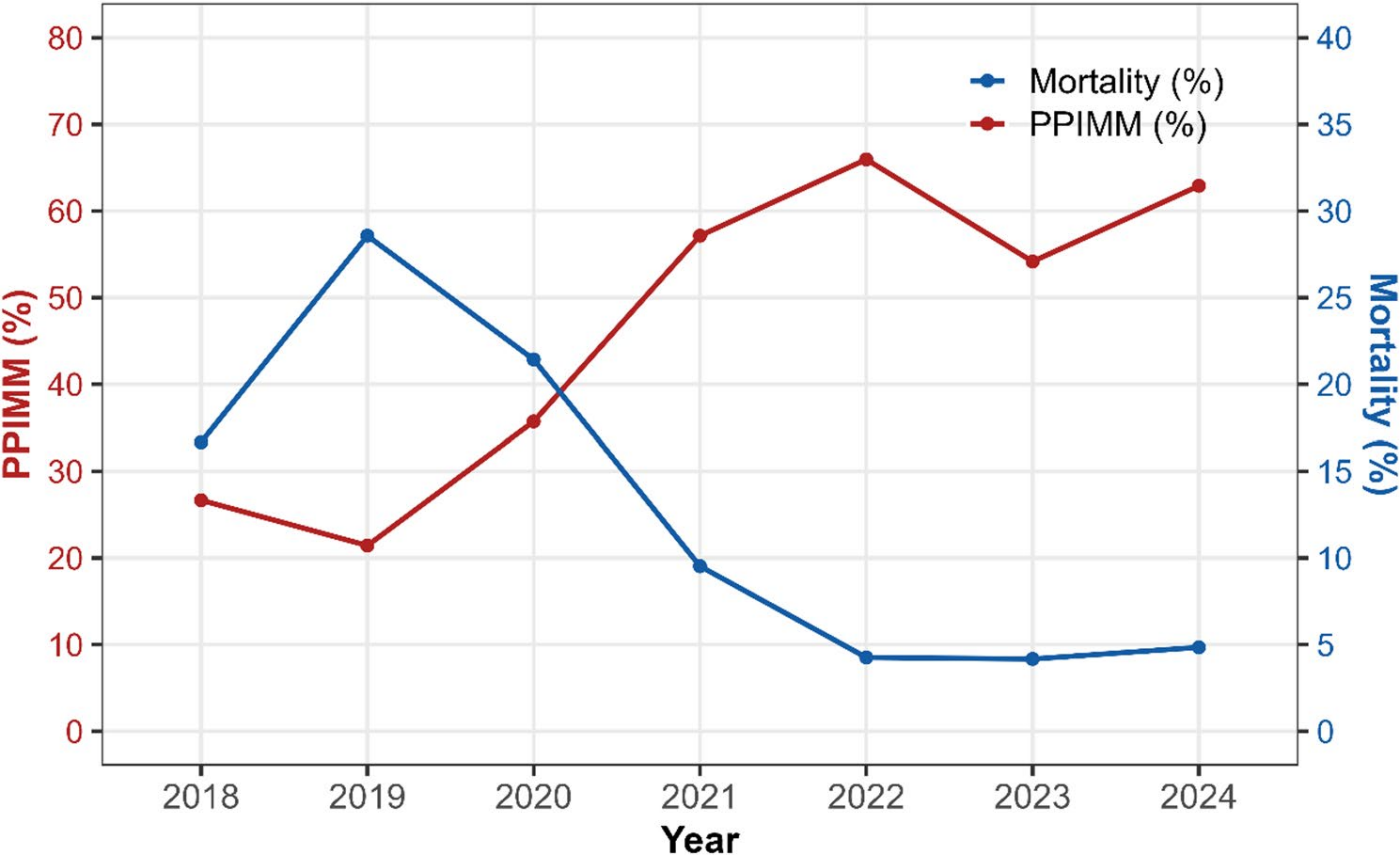
Changes in the proportion of neonates managed with the PPIMM and operative mortality rates from 2018 to 2024. Abbreviations: PPIMM, Prenatal–postnatal integrated management model

Between 2018 and 2024, the proportion of neonates managed with PPIMM increased overall, rising from approximately 27% in 2018 to a peak of 66% in 2022 (Fig. 5). During this period, the operative mortality rate demonstrated an overall downward trend, ultimately reaching about 5% in 2024 despite annual fluctuations. Annual distribution and the proportion of PPIMM versus non-PPIMM procedures are shown in Supplementary Fig. 4. Comparisons between periods showed that, in 2022–2024, operative mortality (4.42% vs. 19.35%, *P* < 0.001) was significantly lower than in 2018–2021 (Table 3). Notably, the incidence of prematurity was higher in 2022–2024 (13.26% vs. 3.23%, *P* = 0.009), and both age at surgery (11.57 ± 7.85 vs. 13.77 ± 7.54 days, *P* = 0.025) and weight at surgery (3.11 ± 0.56 vs. 3.39 ± 0.60 kg, *P* < 0.001) were lower compared with the earlier period. There were no significant differences between the two groups in unplanned reoperation, late mortality, STAT category, emergent procedures, sex, twin pregnancy, or in vitro fertilization. In the multivariable Cox regression, after adjusting for surgical period, the time factor was not statistically significant (*P* = 0.231), indicating a limited independent impact on mortality risk (Supplementary Table 8).

**Table 3:** Perioperative characteristics and clinical outcomes between 2018–2021 and 2022–2024

Variable	2018–2021(*n* = 93)	2022–2024(*n* = 181)	*P* value
PPIMM, *n* (%)	31 (33.33%)	109 (60.22%)	< 0.001
Male sex, *n* (%)	63 (67.74%)	120 (66.3%)	0.917
Premature infant, *n* (%)	3 (3.23%)	24 (13.26%)	0.009
Twin pregnancy, *n* (%)	5 (5.38%)	21 (11.6%)	0.148
In Vitro Fertilization, *n* (%)	7 (7.53%)	13 (7.18%)	1.000
Age at surgery, days	13.77 ± 7.54	11.57 ± 7.85	0.025
Weight at surgery, kg	3.39 ± 0.60	3.11 ± 0.56	< 0.001
Preoperative arrhythmia, *n* (%)	4 (4.3%)	5 (2.76%)	0.494
Preoperative ventilation mode, *n* (%)			0.946
Spontaneous breathing	66 (70.97%)	126 (69.61%)	-
Non-Invasive ventilation	12 (12.9%)	26 (14.36%)	-
Endotracheal intubation	15 (16.13%)	29 (16.02%)	-
Preoperative cardiopulmonary resuscitation, *n* (%)	10 (10.75%)	15 (8.29%)	0.653
Preoperative vasoactive drug use, *n* (%)	17 (18.28%)	44 (24.31%)	0.326
Preoperative prostaglandin E1 use, *n* (%)	39 (41.94%)	67 (37.02%)	0.509
STAT, *n* (%)			0.960
STAT-1	1 (1.08%)	3 (1.66%)	-
STAT-2	32 (34.41%)	57 (31.49%)	-
STAT-3	21 (22.58%)	44 (24.31%)	-
STAT-4	39 (41.94%)	75 (41.44%)	-
STAT-5	0 (0%)	2 (1.10%)	-
Emergent procedure, *n* (%)	13 (13.98%)	14 (7.73%)	0.153
Reintubation, *n* (%)	10 (10.75%)	10 (5.52%)	0.184
ECMO, *n* (%)	3 (3.23%)	7 (3.87%)	1.000
Peritoneal dialysis, *n* (%)	13 (13.98%)	34 (18.78%)	0.407
Postoperative ICU stay, days	11.00 (8.00–16.00)	10.00 (7.00–14.00)	0.187
Operative mortality, *n* (%)	18 (19.35%)	8 (4.42%)	< 0.001
Unplanned reoperation during hospitalization, *n* (%)	8 (8.60%)	15 (8.28%)	0.929
Late mortality, *n* (%)	6 (6.45%)	5 (2.76%)	0.251
Reoperation after discharge, *n* (%)	2 (2.15%)	11 (6.08%)	0.230

*Abbreviations*: *ECMO* Extracorporeal membrane oxygenation, *CPB* Cardiopulmonary bypass, *ACC* Aortic cross-clamp, *PPIMM* Prenatal–postnatal integrated management model, *STAT* the Society of Thoracic Surgeons–European Association for Cardio-Thoracic Surgery

## Discussion

This study found that PPIMM was associated with earlier surgical intervention, better preoperative stability, and improved survival compared with the non-PPIMM group. In selected patients, immediate postnatal surgery appeared feasible and was not associated with increased mortality, supporting its potential applicability in clinical practice. Additionally, PPIMM and prenatal diagnosis were identified as protective factors, while preoperative intubation, postoperative ECMO use, and elevated lactate level at 24 h postoperatively were identified as risk factors in neonates with CCHD.

In this study, the implementation of PPIMM was associated with lower neonatal mortality among infants with CCHD. The overall operative mortality in our cohort was 9.4%, which is comparable to rates reported by other high-volume international centers [5, 15]. Notably, the operative mortality rate in the PPIMM group was 5.7%. While this rate appears lower than those reported in some previous studies, direct comparisons should be interpreted with caution due to differences in patient characteristics such as age, weight, and STAT category distribution. Patients in STAT category 5 accounted for less than 1% of our cohort, which may partially explain the relatively low operative mortality observed. Although TAPVC was more frequent in the non-PPIMM group, mortality within the TAPVC subgroup was similar between groups, suggesting that differences in disease distribution may have had a limited influence on outcomes. Moreover, STAT category distribution, CPB time, and ACC time were similar between the PPIMM and non-PPIMM groups, suggesting that the outcome differences are unlikely to be driven by operative complexity or time alone, though unmeasured surgical factors may still contribute. Overall improvements in medical care, increased case volume, and greater PPIMM proportion in recent years could partly contribute to the observed reduction in mortality. However, multivariable analysis indicated that the independent effect of time period was limited. After adjustment for confounding factors, PPIMM remained significantly associated with lower operative mortality, indicating a potential benefit that warrants further validation in prospective, multicenter studies.

In neonates with a prenatal diagnosis, PPIMM was associated with earlier admission and earlier surgery. Timely intervention in neonatal CCHD is typically associated with more favorable outcomes. Although operative mortality was lower in the PPIMM group than in the prenatally diagnosed non-PPIMM group, the difference was not statistically significant and, given the small number of deaths and the imbalance in group sizes, these results should be interpreted with caution. The beneficial effect of PPIMM on prognosis likely reflects a combination of factors, including higher rates of prenatal diagnosis, better preoperative condition, and earlier admission and intervention. PPIMM enables earlier identification and assessment of patients, thereby supporting the formulation of individualized management strategies for each neonate. Depending on disease severity and individualized risk stratification, interventions may include in-utero procedures, immediate postnatal surgery, or early neonatal surgical repair. These findings support further evaluation of PPIMM, which may inform its broader implementation to improve outcomes for neonates with CCHD.

In this selected cohort, immediate postnatal surgery appeared feasible and was not associated with increased mortality. Previous reports have also described immediate postnatal surgery as feasible and associated with acceptable short- and mid-term outcomes in selected neonates with CCHD. Moray et al. reported that in neonates at high risk for acute cardiorespiratory instability shortly after birth, such as those with transposition of the great arteries with intact ventricular septum or obstructed total anomalous pulmonary venous connection, up to 50% of infants may experience hemodynamic deterioration or need for intervention within the first two hours of life [16]. One of the aims of immediate postnatal surgery is to provide timely intervention before the onset of hemodynamic compromise. Hogue et al. reported eight neonates with obstructed TAPVC who underwent immediate postnatal surgery within one hour of birth, with an overall survival rate of 88% (*n* = 7/8) over a median follow-up of 5.7 years [17]. Another aim of immediate postnatal surgery is to promote early cardiac development. In neonates with pulmonary atresia with intact ventricular septum and right ventricular hypoplasia, establishing antegrade pulmonary blood flow immediately after birth may provide early volume loading to the right ventricle, which may promote right ventricular development and potentially improving long-term outcomes. Currently, evidence supporting immediate postnatal surgery remains limited, and further prospective studies are needed to clarify its long-term effects. The decision to perform immediate postnatal surgery should be made with caution, requiring thorough assessment, careful planning, and seamless multidisciplinary coordination to minimize risks and improve outcomes.

This study suggests that prenatal diagnosis was associated with lower mortality among neonates with CCHD. The rate of prenatal diagnosis reached 100% in the PPIMM group, compared to only 52.9% in the non-PPIMM group. Previous studies have reported conflicting results regarding the effect of prenatal diagnosis on mortality. Dischinger et al. found that patients who received a prenatal diagnosis experienced higher mortality [18]. In contrast, Zhang et al. [6] reported that prenatal diagnosis improved preoperative condition and reduced one-year mortality, which is consistent with our findings. Notably, in the non-PPIMM group, prenatal diagnosis was associated with a lower mortality rate compared with postnatal diagnosis, and was confirmed by multivariable Cox regression analysis. The observed survival benefit may be attributed to earlier disease recognition and more structured peri-delivery planning. These results suggest that, even in resource-limited and remote areas where PPIMM cannot be fully implemented, efforts should be made to increase the rate of prenatal diagnosis. These findings underscore the need to improve fetal CHD screening and diagnosis to enable earlier intervention and better outcomes in neonates with CCHD.

The PPIMM group had lower rates of preoperative intubation and emergency procedures, reflecting a more favorable preoperative physiological status. This may be attributed to earlier hospital admission and individualized supportive care facilitated by the PPIMM. Reddy et al. reported that poor preoperative physiological status increases the risk of adverse postoperative outcomes [19]. Jacobs et al. identified preoperative intubation as a risk factor for mortality [20], and Miyata et al. demonstrated that emergency surgery is a strong predictor of both 30-day and 90-day mortality [21]. In our study, the lower prevalence of these risk factors in the PPIMM group suggests a potential advantage of this model, underscoring the importance of optimizing preoperative condition to improve outcomes in neonates with CCHD.

This study demonstrated that elevated lactate levels and postoperative ECMO use were both significantly associated with mortality in neonates with CCHD. Elevated lactate serves as a marker of tissue hypoxia and inadequate microcirculatory perfusion. Zhang et al. reported a lactate level threshold of 4.8 mmol/L at 24 h postoperatively [22], which was similar to that observed in our study. These thresholds indicate levels above which the risk of mortality increases substantially, underscoring their potential utility for early postoperative risk stratification and timely intervention. Valencia et al. identified elevated lactate as an independent risk factor for postoperative mortality in neonates with CHD [23], supporting its value as a readily measurable biomarker in clinical decision-making. The use of ECMO is generally indicative of critical postoperative deterioration and severe cardiac dysfunction, and Zhang et al. reported its strong association with mortality in neonates with CHD [22]. These findings are consistent with our results and reinforce the importance of close monitoring of lactate levels, postoperative cardiac function, and circulatory status to improve outcomes.

This study has several limitations. First, this was a single-center retrospective study, which may limit the generalizability of the findings and introduce potential institutional bias. Second, the higher proportion of TAPVC cases in the non-PPIMM group may have partially influenced outcome comparisons. Third, the limited sample size and low event rates, particularly in subgroup analyses, may have reduced statistical power. Fourth, long-term neurodevelopmental outcomes were not assessed. Fifth, the long study period may involve temporal changes in surgical techniques and resources that may affect the outcomes. Future prospective, multicenter studies with long-term follow-up are needed to further validate the prognostic value of PPIMM.

## Conclusion

This retrospective cohort study found that PPIMM was associated with earlier admission and surgery, better preoperative status, and lower operative mortality. However, PPIMM did not reduce late mortality or unplanned reoperation rates. In this selected cohort, immediate postnatal surgery appeared feasible and was not associated with increased operative mortality. Additionally, PPIMM and prenatal diagnosis were identified as protective factors, while preoperative intubation, postoperative ECMO use, and elevated lactate level at 24 h postoperatively were identified as risk factors in neonates with CCHD. These findings highlight the need for further promotion and widespread implementation of the PPIMM to improve outcomes for neonates with CCHD.

## Supplementary Information

Supplementary Material 1.

## Data Availability

The datasets used and/or analysed during the current study are available from the corresponding author on reasonable request.

## Abbreviations

PPIMM: Prenatal–postnatal integrated management model
CCHD: Critical congenital heart disease
ECMO: Extracorporeal membrane oxygenation
CPB: Cardiopulmonary bypass
ACC: Aortic cross-clamp
STAT: Society of Thoracic Surgeons–European Association for Cardio-Thoracic Surgery Congenital Heart Surgery Category
VIS: Vasoactive–inotropic score
TGA: Transposition of the great arteries
TAPVC: Total anomalous pulmonary venous connection
IAA: Interrupted aortic arch
COA: Coarctation of the aorta
PA: Pulmonary atresia
TOF: Tetralogy of fallot
