# Supplementary material for: Prenatal-postnatal integrated management model improves outcomes of neonatal cardiac surgery in critical congenital heart disease: a retrospective cohort study
Source: BMC Pediatr. 2026 Jan 5;26:6. doi: 10.1186/s12887-025-06378-x (PMC12771998; doi:10.1186/s12887-025-06378-x)
Supplement: Supplementary file 1 — Supplementary Material 1. [file 12887_2025_6378_MOESM1_ESM.pdf]

Prenatal-Postnatal Integrated Management Model Improves Outcomes of Neonatal Cardiac Surgery in Critical Congenital Heart Disease: A Retrospective Cohort Study

Supplementary Materials

Supplementary Figure 1. Perioperative management pathway for neonates with critical congenital heart disease in the PPIMM and non-PPIMM groups

Supplementary Figure 2. Perioperative management pathway for immediate postnatal surgery in neonates with critical congenital heart disease

Supplementary Figure 3. Kaplan-Meier survival curves comparing prenatal diagnosis and postnatal diagnosis subgroups within the non-PPIMM group

Supplementary Figure 4. Annual distribution and proportions of PPIMM vs non-PPIMM procedures among neonates with CCHD (January 2018–August 2024)

Supplementary Table 1. Distinctions in Perioperative Management Pathways Between PPIMM and Non-PPIMM Groups

Supplementary Table 2. Distribution of disease categories in the PPIMM and non-PPIMM groups

Supplementary Table 3. Perioperative characteristics in the prenatal-diagnosed subset (PPIMM vs non-PPIMM).

Supplementary Table 4. Preoperative and postoperative characteristics of TAPVC patients in the PPIMM and non-PPIMM groups

Supplementary Table 5. Clinical characteristics of neonates undergoing immediate postnatal surgery

Supplementary Table 6. Perioperative characteristics by timing of diagnosis (prenatal vs postnatal) in the non-PPIMM group.

Supplementary Table 7. Univariable Cox regression analyses of mortality in the Total cohort, PPIMM group, and non-PPIMM group

Supplementary Table 8. Multivariable Cox regression analyses of mortality adjusted for surgical period (2018–2021 vs. 2022–2024)

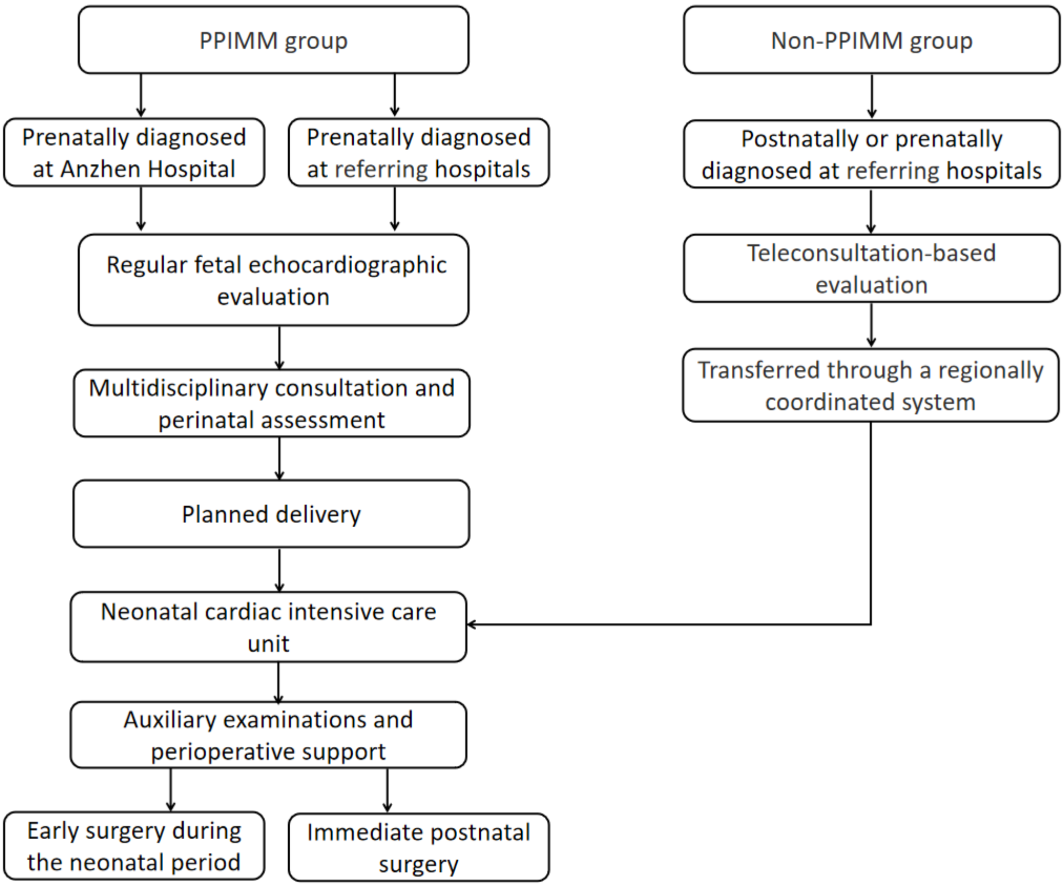

Supplementary Figure 1. Perioperative management pathways for neonates with critical congenital heart disease in the PPIMM and non-PPIMM groups. Abbreviations: PPIMM, Prenatal–postnatal integrated management model.

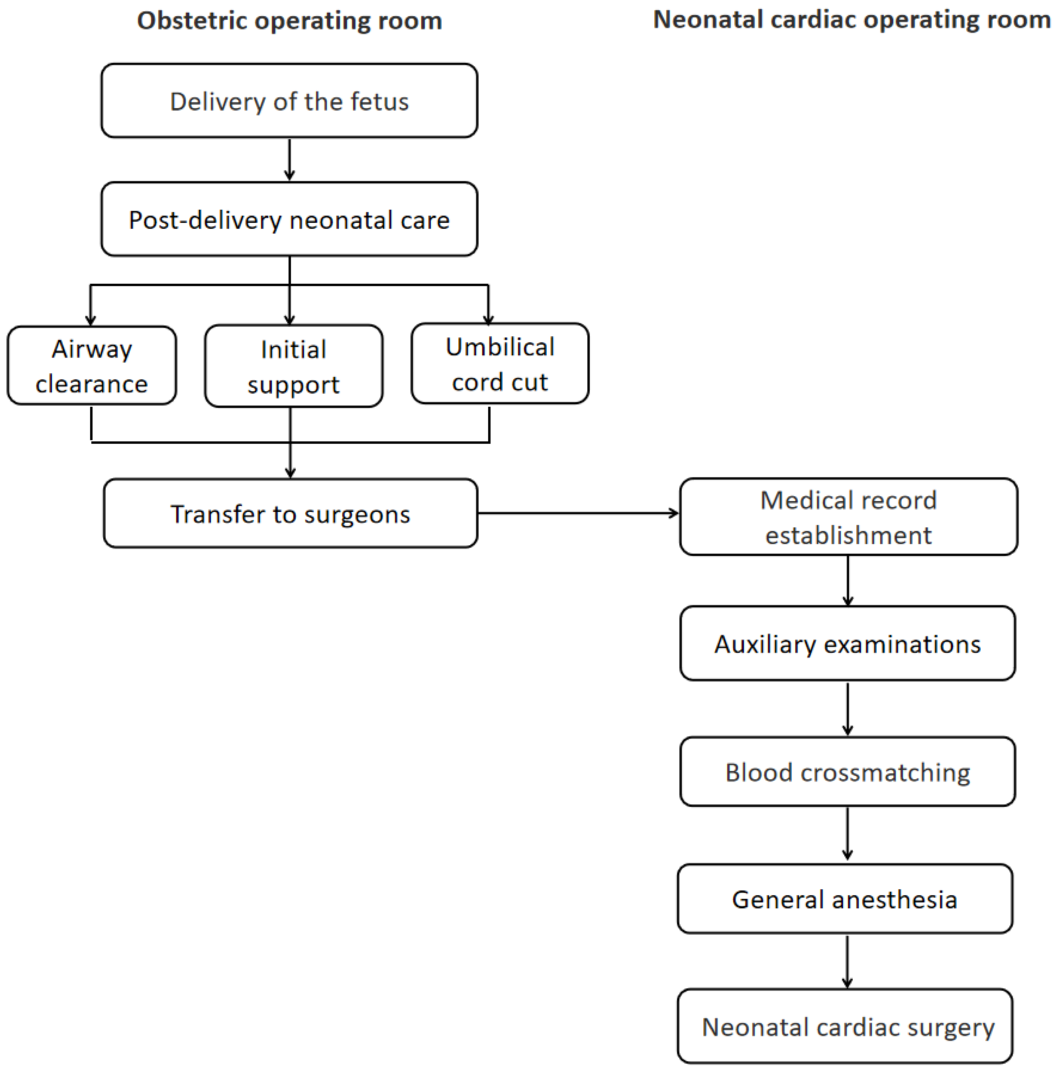

Supplementary Figure 2. Perioperative management pathway for immediate postnatal surgery in neonates with critical congenital heart disease.

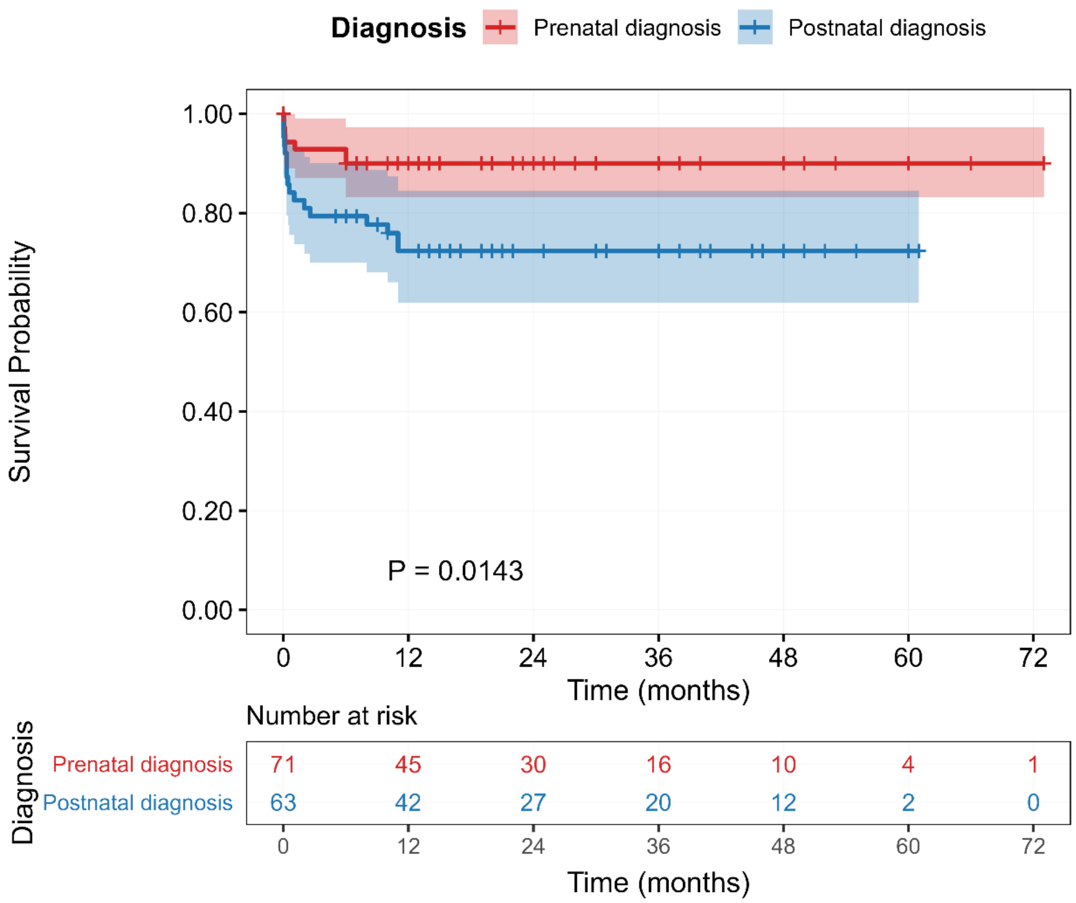

Supplementary Figure 3. Kaplan-Meier survival curves comparing prenatal diagnosis and postnatal diagnosis subgroups within the non-PPIMM group. Abbreviations: PPIMM, Prenatal–postnatal integrated management model.

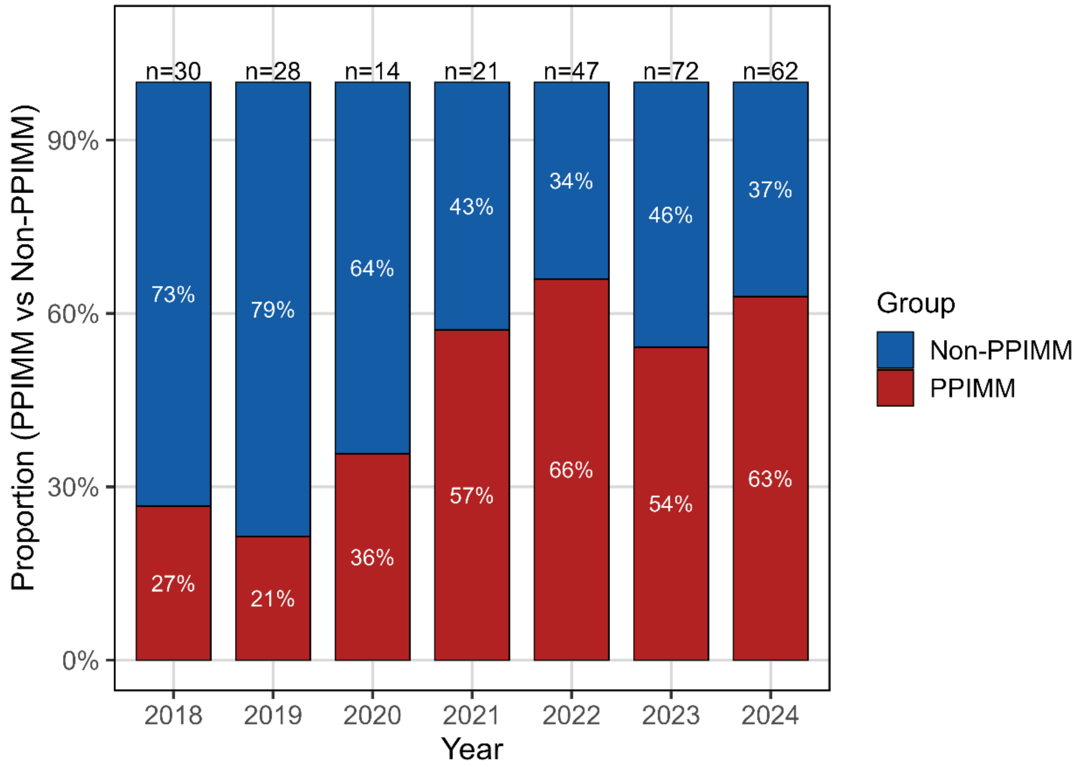

Supplementary Figure 4. Annual distribution and proportions of PPIMM vs non-PPIMM procedures among neonates with CCHD (January 2018–August 2024). Abbreviations: PPIMM, Prenatal–postnatal integrated management model.

Supplementary Table 1. Distinctions in Perioperative Management Pathways Between PPIMM and Non-PPIMM Groups

**Management component**	**PPIMM group**	**Non-PPIMM group**
Diagnosis timing	Prenatal diagnosis	Prenatal or postnatal diagnosis
Prenatal monitoring	Serial fetal echocardiography at Beijing Anzhen Hospital	Absent or inconsistent across referring hospitals
Prenatal planning	Multidisciplinary consultation and develop individualized perinatal management plans	Absent or unstructured multidisciplinary prenatal planning
Delivery plan	Planned delivery at Beijing Anzhen Hospital	Delivery at referring hospitals, followed by inter-hospital transfer
Immediate postnatal care	Prompt neonatal cardiac ICU admission; standardized preoperative evaluation (echocardiography, ECG, chest X-ray, CT as indicated)	Prompt neonatal cardiac ICU admission after transfer; same evaluation protocol
Supportive interventions	Oxygen, vasoactive agents, diuretics, prostaglandin E_1 (ductus-dependent CCHD), and other measures as needed	Same supportive interventions after arrival
Surgical timing	In-utero procedures, immediate postnatal surgery, or early neonatal surgery	Early neonatal surgery after transfer and evaluation
Surgical decision-making	Based on anatomy, physiology, and hemodynamic assessment	Based on anatomy, physiology, and hemodynamic assessment

Abbreviations: CCHD, Critical congenital heart disease; CT, Computed tomography; ECG, Electrocardiography; PPIMM, Prenatal–postnatal integrated management model.

Supplementary Table 2. Distribution of disease categories in the PPIMM and non-PPIMM groups.

Variable	PPIMM group （n=140）	Non-PPIMM group（n=134）	*P* value
COA	17(12.14%)	27(20.15%)	0.101
DORV	5(3.57%)	3(2.24%)	0.767
IAA	11(7.86%)	6(4.48%)	0.364
TAPVC	21(15.00%)	36(26.87%)	0.023
PA	19(13.57%)	16(11.94%)	0.823
TGA	24(17.14%)	19(14.18%)	0.611
SPS	10(7.14%)	10(7.46%)	1.000
TOF	29(20.71%)	16(11.94%)	0.072
PTA	1(0.71%)	0(0.00%)	1.000
AOPA	2(1.43%)	1(0.75%)	1.000
HLHS	1(0.71%)	0(0.00%)	1.000

Abbreviations: PPIMM, Prenatal–postnatal integrated management model; CoA, Coarctation of the aorta; DORV, Double outlet right ventricle; IAA, Interrupted aortic arch; TAPVC, Total anomalous pulmonary venous connection; PA, Pulmonary atresia; TGA, Transposition of the great arteries; SPS, Severe pulmonary stenosis; TOF, Tetralogy of fallot; PTA, Persistent truncus arteriosus; AOPA, Anomalous origin of pulmonary artery From ascending aorta; HLHS, Hypoplastic left heart syndrome.

Supplementary Table 3. Perioperative characteristics in the prenatal-diagnosed subset (PPIMM vs non-PPIMM).

Variable	PPIMM (n=140)	Non-PPIMM with prenatal diagnosis (n=71)	P value
Male sex, n (%)	97 (69.29%)	45 (63.38%)	0.478
Premature infant, n (%)	17 (12.14%)	6 (8.45%)	0.562
Age at admission, days	0.00 (0.00–0.00)	9.00 (3.00–15.00)	<0.001
Age at surgery, days	8.00 (3.00–14.00)	16.00 (10.00–20.00)	<0.001
Preoperative ventilation mode, n (%)			0.261
Spontaneous breathing	103 (73.57%)	48 (67.61%)	-
Non-invasive ventilation	21 (15.00%)	9 (12.68%)	-
Endotracheal intubation	16 (11.43%)	14 (19.72%)	-
Preoperative cardiopulmonary resuscitation, n (%)	10 (7.14%)	6 (8.45%)	0.949
STAT, n (%)			0.942
STAT-1	2 (1.43%)	0 (0.00%)	-
STAT-2	47(33.57%)	24 (33.80%)	-
STAT-3	34(24.29%)	19 (26.76%)	-
STAT-4	56 (40.00%)	27 (38.03%)	-
STAT-5	1 (0.71%)	1 (1.41%)	-
Emergent procedure, n (%)	8 (5.71%)	7 (9.86%)	0.410
CPB time, min	140.00 (106.00–165.00)	138.00 (113.00–186.00)	0.174
ACC time, min	85.50 (65.00–110.25)	86.00 (64.00–114.00)	0.790
ECMO, n (%)	4 (2.86%)	1 (1.41%)	0.665
Peritoneal dialysis, n (%)	21 (15.00%)	15 (21.13%)	0.355
Operative mortality, n (%)	8 (5.71%)	6 (8.45%)	0.644
Follow up period, months	20.70(9.00–30.30)	19.00 (7.00–30.00)	0.718

Abbreviations: ECMO, Extracorporeal membrane oxygenation; CPB, Cardiopulmonary bypass; ACC, Aortic cross-clamp; PPIMM, Prenatal–postnatal integrated management model; STAT, Society of Thoracic Surgeons–European Association for Cardio-Thoracic Surgery.

Supplementary Table 4. Preoperative and postoperative characteristics of TAPVC patients in the PPIMM and non-PPIMM groups

**Variable**	**PPIMM group (n = 21)**	**Non-PPIMM group (n = 36)**	***P* value**
**TAPVC type, n (%)**			0.520
Supracardiac	13 (61.90%)	16 (44.44%)	
Cardiac	4 (19.05%)	10 (27.78%)	
Infracardiac	3 (14.29%)	9 (25.00%)	
Mixed	1 (4.76%)	1 (2.78%)	
Obstructed TAPVC, n (%)	8 (38.10%)	14 (38.89%)	1.000
Male sex, n (%)	12 (57.14%)	26 (72.22%)	0.261
Age at surgery, days	4.00[1.00-10.00]	13.50[8.00-21.00]	< 0.001
Weight at surgery, kg	3.10[2.90-3.50]	3.50[3.20-3.80]	0.008
Operative mortality, n (%)	2 (9.52%)	6 (16.67%)	0.696
Late mortality, n (%)	3 (14.29%)	4 (11.11%)	0.700

Abbreviations: PPIMM, Prenatal–postnatal integrated management model; TAPVC, Total anomalous pulmonary venous connection.

Supplementary Table 5. Clinical characteristics of neonates undergoing immediate postnatal surgery

Variable	Value (Mean ± SD / Median [IQR])
Male, n (%)	8 (66.66%)
Premature infant, n (%)	1 (8.33%)
Birth weight (kg)	3.00 ± 0.50
Preoperative O_2 saturation (%)	87.50 [85.00–90.00]
Preoperative lactate (mmol/L)	1.50 ± 0.44
CPB time, min	165.00 (136.50–172.25)
ACC time, min	90.00 (75.75–113.50)
Reintubation, n (%)	1 (8.33%)
Delayed sternal closure, n (%)	1 (8.33%)
Peritoneal dialysis, n (%)	1 (8.33%)
Postoperative mechanical ventilation (hours)	60.50 (42.25–120.13)
Postoperative ICU stay (days)	9.00 (6.50–10.00)
Operative mortality, n (%)	0
Unplanned reoperation, n (%)	0
Follow-up time (months)	13.00 (9.00–15.00)
Late mortality, n (%)	0

Abbreviations: ECMO, Extracorporeal membrane oxygenation; CPB, Cardiopulmonary bypass; ACC, Aortic cross-clamp.

Supplementary Table 6. Perioperative characteristics by timing of diagnosis (prenatal vs postnatal) in the non-PPIMM group.

**Variable**	**Prenatal diagnosis （n=71）**	**Postnatal diagnosis （n=63）**	**P value**
Male sex, n (%)	45 (63.38%)	41 (65.08%)	0.981
Premature infant, n (%)	6 (8.45%)	4 (6.35%)	0.749
Cesarean section, n (%)	46 (64.79%)	38 (60.32%)	0.722
Twin pregnancy, n (%)	10 (14.08%)	2 (3.17%)	0.034
In Vitro Fertilization, n (%)	4 (5.63%)	2 (3.17%)	0.684
Age at admission, days	9.00 (3.00–15.00)	11.00 (4.00–17.00)	0.206
Age at surgery, days	16.00 (10.00–20.00)	16.00 (9.50–21.00)	0.829
Preoperative arrhythmia, n (%)	1 (1.41%)	2 (3.17%)	0.601
Preoperative ventilation mode, n (%)			0.936
Spontaneous breathing	48 (67.61%)	41 (65.08%)	-
Non-Invasive ventilation	9 (12.68%)	8 (12.70%)	-
Endotracheal intubation	14 (19.72%)	14 (22.22%)	-
Preoperative cardiopulmonary resuscitation, n (%)	6 (8.45%)	9 (14.29%)	0.427
Preoperative vasoactive drug use, n (%)	16 (22.54%)	13 (20.63%)	0.955
Preoperative prostaglandin E1 use, n (%)	25 (35.21%)	20 (31.75%)	0.810
**STAT, n (%)**			0.254
STAT-1	0 (0.00%)	2 (3.17%)	-
STAT-2	24 (33.80%)	18 (28.57%)	-
STAT-3	19 (26.76%)	12 (19.05%)	-
STAT-4	27 (38.03%)	31 (49.21%)	-
STAT-5	1 (1.41%)	0 (0.00%)	-
Emergent procedure, n (%)	7 (9.86%)	12 (19.05%)	0.203
ACC time, min	86.00 (64.00–114.00)	76.00 (49.50–102.00)	0.163
CPB time, min	138.00 (113.00-186.00)	144.00 (91.00–182.00)	0.576
Reintubation, n (%)	3 (4.23%)	7 (11.11%)	0.189
ECMO, n (%)	1 (1.41%)	5 (7.94%)	0.099
Operative mortality, n (%)	6 (8.45%)	12 (19.05%)	0.123
Follow up period, months	19.00 (7.00–30.00)	20.00 (7.50–45.50)	0.591
Late mortality, n (%)	0 (0.00%)	6 (9.52%)	0.009

Abbreviations: Abbreviations: ECMO, Extracorporeal membrane oxygenation; CPB, Cardiopulmonary bypass; ACC, Aortic cross-clamp; PPIMM, Prenatal–postnatal integrated management model; STAT, Society of Thoracic Surgeons–European Association for Cardio-Thoracic Surgery.

Supplementary Table 7. Univariable Cox regression analyses of operative mortality in the Total cohort, PPIMM group, and non-PPIMM group

Variable	Total Cohort, HR (95% CI), *P* value	PPIMM Group, HR (95% CI), *P* value	Non-PPIMM Group, HR (95% CI), *P* value
Preoperative intubation	5.06(2.34–10.94), < 0.001	4.93 (1.18–20.65), 0.029	3.23 (1.27–8.20), 0.013
Preoperative cardiopulmonary resuscitation	2.53 (0.95–6.72), 0.062	2.05 (0.42–8.76), 0.350	3.50 (1.25–9.84), 0.017
Preoperative vasoactive drug use	1.89 (0.84–4.23), 0.124	5.88 (1.40–24.60), 0.015	1.02 (0.37–3.10), 0.970
Preoperative Prostaglandin E1 use	2.65 (1.20–5.85), 0.016	9.48 (1.17–77.07), 0.035	2.07 (0.82–5.22), 0.123
Emergent surgery	3.76 (1.58–8.95), 0.003	2.42 (0.3–19.65), 0.409	3.50 (1.31–9.35), 0.012
Delayed sternal closure	1.74 (0.73–4.15), 0.209	1.83 (0.10–6.76), 0.863	1.94 (0.73–5.18), 0.184
ECMO use	11.36 (5.05–20.56), < 0.001	11.19 (2.24–55.79), 0.003	10.07 (3.76–26.97), < 0.001
Reintubation	8.75 (3.97–19.31), < 0.001	8.58 (2.05–35.93), 0.003	9.76 (3.77–25.31), < 0.001
Maximum VIS within 24 hours postoperatively	1.06 (1.04–1.09), < 0.001	1.06 (1.03–1.10), 0.001	1.09 (1.05–1.14), < 0.001
Lactate level at ICU admission	1.20 (1.14–1.26), < 0.001	1.20 (1.11–1.30), < 0.001	1.29 (1.17–1.42), < 0.001
Lactate level at 24 hours postoperatively	1.13 (1.09–1.18), < 0.001	1.15 (1.08–1.22), < 0.001	1.14 (1.08–1.19), < 0.001

Abbreviations: ECMO, Extracorporeal membrane oxygenation; PPIMM, Prenatal–postnatal integrated management model; VIS, Vasoactive–inotropic score.

Supplementary Table 8 Multivariable Cox regression analyses of mortality adjusted for surgical period (2018–2021 vs. 2022–2024) in the Total cohort

Variable	HR	95% CI	P value
PPIMM	0.43	0.18–0.92	0.045
Preoperative intubation	2.70	1.12–6.48	0.027
Postoperative ECMO use	5.16	2.00–13.29	<0.001
Lactate at 24h postoperatively	1.11	1.06–1.16	<0.001
Surgical period (2022–2024 vs. 2018–2021)	0.59	0.25–1.39	0.231

Abbreviations: HR, Hazard ratio; CI, Confidence interval; ECMO, Extracorporeal membrane oxygenation. Note: The model was adjusted for surgical period to control for potential confounding from temporal improvements in overall medical care.
